# *Chenopodium ambrosioides* L. Reduces Synovial Inflammation and Pain in Experimental Osteoarthritis

**DOI:** 10.1371/journal.pone.0141886

**Published:** 2015-11-02

**Authors:** Gustavo P. Calado, Alberto Jorge O. Lopes, Livio M. Costa Junior, Francisco das Chagas A. Lima, Lucilene A. Silva, Wanderson S. Pereira, Flávia M. M. do Amaral, João Batista S. Garcia, Maria do Socorro de S. Cartágenes, Flávia R. F. Nascimento

**Affiliations:** 1 Health Sciences Graduate Program, Federal University of Maranhao, Biologic and Health Sciences Center, Av. dos Portugueses 1966, São Luís, MA, CEP:65085–580, Brazil; 2 Chemistry Graduate Program, Quantum Chemistry Computational Group, Department of Chemistry, State University of Piauí – 64002–150, Teresina, PI, Brazil; Faculté de médecine de Nantes, FRANCE

## Abstract

The chronicity of osteoarthritis (OA), characterized by pain and inflammation in the joints, is linked to a glutamate receptor, N-methyl-D-aspartate (NMDA). The use of plant species such as *Chenopodium ambrosioides* L. (Amaranthaceae) as NMDA antagonists offers a promising perspective. This work aims to analyze the antinociceptive and anti-inflammatory responses of the crude hydroalcoholic extract (HCE) of *C*. *ambrosioides* leaves in an experimental OA model. Wistar rats were separated into six groups (n = 24): clean (C), negative control (CTL-), positive control (CTL+), HCE0.5, HCE5 and HCE50. The first group received no intervention. The other groups received an intra-articular injection of sodium monoiodoacetate (MIA) (8 mg/kg) on day 0. After six hours, they were orally treated with saline, Maxicam plus (meloxicam + chondroitin sulfate) and HCE at doses of 0.5 mg/kg, 5 mg/kg and 50 mg/kg, respectively. After three, seven and ten days, clinical evaluations were performed (knee diameter, mechanical allodynia, mechanical hyperalgesia and motor activity). On the tenth day, after euthanasia, synovial fluid and draining lymph node were collected for cellular quantification, and cartilage was collected for histopathological analysis. Finally, molecular docking was performed to evaluate the compatibility of ascaridole, a monoterpene found in HCE, with the NMDA receptor. After the third day, HCE reduced knee edema. HCE5 showed less cellular infiltrate in the cartilage and synovium and lower intensities of allodynia from the third day and of hyperalgesia from the seventh day up to the last treatment day. The HCE5 and HCE50 groups improved in forced walking. In relation to molecular docking, ascaridole showed NMDA receptor binding affinity. *C*. *ambrosioides* HCE was effective in the treatment of OA because it reduced synovial inflammation and behavioral changes due to pain. This effect may be related to the antagonistic effect of ascaridole on the NMDA receptor.

## Introduction

Many plants are popularly used for nutritional and/or therapeutic purposes and specifically in the development and discovery of new drugs for the treatment of degenerative diseases such as osteoarthritis (OA). The need for scientific evidence has stimulated validation studies of plant species such as *Chenopodium ambrosioides* L. (Amaranthaceae), which has been popularly used in diuretic, anti-inflammatory and healing capacities [1]. This plant species is an annual or perennial shrub with strong aromatic odor. It is found throughout the Brazilian territory and is known in Brazil as "mastruz" or "Erva-de-Santa-Maria" [2]. It is a plant rich in monoterpenes, including ascaridole, cited as one of its most abundant compounds [3].

Some biological actions of this plant have been scientifically demonstrated, especially in studies using extracts from its leaves. Some of these studies have involved modulation of the immune and inflammatory responses, for example, soft tissue and bone repair [4]; antitumor [5], antileishmanial [6–7], analgesic [8], anti-inflammatory and antinociceptive [9] actions; and in the treatment of *Helicobacter pylori* [10].

Although the anti-inflammatory effects of *C*. *ambrosioides* have been demonstrated in different experimental models, there is still no evidence of the effect of this species on clinical OA, associating these aspects with immunopharmacological evaluations. Furthermore, to date, there is little evidence of possible substances involved in the anti-inflammatory and antinociceptive effects reported in the literature.

The ascaridole, a bicyclic monoterpene, is one of the most abundant terpenoid in *Chenopodium* genus [3]. Recent studies have suggested that ascaridole found in ethanolic extracts of *C*. *ambrosioides* leaves, may be primarily responsible for its antinociceptive, sedative and anti-inflammatory effects [9,11,12]. These findings led us to use molecular docking tests [13] to investigate whether there is an association between ascaridole and specific pain receptors, such as N-methyl-D-aspartate (NMDA). This receptor has been thought responsible for the processes of central sensitization and chronic pain [14]. Some NMDA receptor antagonists have demonstrated the capacities to reduce chronic pain and to prevent hyperalgesic phenomena.

Our data demonstrated that treatment with *C*. *ambrosioides* promotes analgesic and anti-inflammatory effects, improving the clinical aspects of experimental OA, and that this effect is likely associated with the antagonistic action of ascaridole on the NMDA receptor.

## Materials and Methods

### Animals

Two months-old Wistar male rats (*Rattus norvegicus*, albinus variety), were bred in our animal facilities at the Federal University of Maranhao (UFMA), under standard conditions. All procedures described were reviewed and approved by the Ethics Committee of the UFMA, in accordance with COBEA (Brazilian College of Animal Experimentation), protocol.23115-006307/2010-2.

### Plant material

The *C*. *ambrosioides* leaves were collected from the Canaã garden in the municipality of Paço do Lumiar–MA (2°30'08.1"S 44°08'39.2"W), and the botanical identification was performed at the Ático Seabra Herbarium of UFMA, with specimen number 1148/SLS017213. A total of 4.8 kg of fresh leaves was collected to obtain the extract, which were then cleaned and dried in an oven at 39°C. The dried material was crushed in a powder mill with a particle size between 250 and 710 μm, resulting in 594 g of powdered raw material, and subjected to percolation using 70% alcohol solution and mixed every eight hours for 24 hours, with 1:5 hydromodule (w/w). The material was passed through a filtration process repeatedly four times and concentrated under reduced pressure to obtain the dry extract [15]. A yield of 16.03% of the crushed dried leaves was obtained.

The crude hydroalcoholic extract (HCE) obtained was evaluated using qualitative and semi-quantitative methods [16]. The results were positive to organic acids, alkaloids, phenolic compounds, saponins, condensed tannins, terpenes, flavononols and flavanones. There were no coumarins, cyanogenic heterosides, resins, hydrolysate tannins, anthocyanins, anthocyanidins, flavones, flavanols, xanthones, chalcones, leucoanthocyanidins and catechins.

### OA model induced by sodium monoiodoacetate (MIA)

The animals were anesthetized by intraperitoneal injection of sodium thiopental 40 mg/kg. After verification of the anesthetic plane, trichotomy of the right hind leg and local asepsis were performed with topical 10% povidone iodine solution. The joint injury was induced by a single intra-articular injection of 2 mg of MIA into this knee, diluted to a maximum volume of 25 μL solution at a dose of 8 mg/kg. With the leg bent at the knee at an angle of approximately 90°, the MIA solution was injected through the patellar ligament using a26GX3/8 needle, in the intra-articular space between the tibia and femur [17].

### Experimental design

On day 0 (zero), the animals received an intra-articular injection of MIA (8 mg/kg) for induction of OA in the knee (n = 25). After the injection, the animals were randomly divided into five groups: negative control (CTL-), positive control (CTL+), HCE0.5, HCE5 and HCE50. After six hours, the groups were treated orally (1x/day) with saline (200 μL), Maxicam Plus 0.5 mg (0.5 mg Meloxicam + 100 mg Chondroitin sulfate) and HCE at doses of 0.5 mg/kg, 5 mg/kg and 50 mg/kg, respectively. A sixth group of animals that received neither MIA nor treatment was created and called Clean.

At three, seven and ten days after the MIA injection, clinical evaluations were performed regarding edema formation, mechanical allodynia, mechanical hyperalgesia and motor activity. On day 10, the animals were euthanized, with an overdose of the anesthetic by intraperitoneal route.

Then, the synovial fluid was collected for cell counts, and the cartilage removed for histopathological analysis. Besides, the draining lymph nodes were collect to cell quantification.

### Clinical evaluations

#### Knee diameter evaluation

To indirectly evaluate the joint inflammation (edema and cellular infiltrate), the knees were measured. A precision tape measure was used to evaluate knee diameter. The tape was positioned medio-laterally in the joint interline region to quantify the joint diameter (cm) of the knee.

#### Evaluation of mechanical allodynia

The evaluation was performed with a digital analgesy-meter (*Insight* Model, São Paulo, Brazil), consisting of a pressure transducer connected to a digital power meter with measurements expressed in grams (g). The device accuracy was 0.1 g. The device was calibrated to record a maximum force of 150 g while maintaining an accuracy of 0.1 g with up to 80 g force. The pressure transducer was connected to the leg of the animal using a 0.5 mm diameter disposable polypropylene tip adapted for this purpose [18]. The test was performed ten consecutive times at intervals of two minutes until three similar readings were taken.

#### Evaluation of mechanical hyperalgesia

An analgesy-meter (*Ugo-Basile*, *Stoelting*, Chicago, IL, USA) was used to perform this test, which generated a linear increase in force (g) on the dorsal surface of the animal's leg until it produced a response characterized as leg withdrawal. Three measurements were taken of the ipsilateral (left) and contralateral (right) legs before and after at regular intervals. The data are expressed as leg withdrawal thresholds in grams [19].

#### Evaluation of motor activity

The animals were placed on a Rotarod, a device similar to a rotor, rotating at a speed of 4 to 40 rpm for a period of 300 seconds. Two basic training tests were performed followed by two recorded evaluations. After induction, three evaluations were performed, and the mean latency was recorded. The animals were allowed a rest period of 15 minutes between evaluations. The data are expressed as walking values and were coded by a single viewer [20].

### Immunopharmacological evaluations

#### Collection of synovial fluid

The synovial fluid was obtained by double washing the joint cavity with 0.2 mL of sodium phosphate buffer (0.15 M, and pH 7.4) containing 37.2 mg of EDTA (0.01 M). The joint lavage was obtained using aspiration and was kept on ice until cell counting [20–21].

#### Histopathological analysis of the synovia cartilage

The synovial tissue was extracted, fixed in 10% formalin for 24 hours and immersed in descaling solution for 15 days. It was then processed using routine methods up to embedding in paraffin blocks. The medial and lateral compartments were separated longitudinally and sent for histological preparation with hematoxylin-eosin staining. Toluidine blue staining was also performed as the cartilage histological control, which specifically stains organic matrix proteoglycans of the cartilage [20–21].

Histological evaluation was performed by subjective interpretation of various parameters, such as inflammatory exudate, impairment of inflammatory exudate (perivascular, interstitial and synovial epithelium), fibrinoid deposits and edema, chosen based on the findings of authors who had used experimental OA models on *Wistar* rats [20–21]. To evaluate these parameters, scores were standardized as follows: 0—absent, 1—light, 2—moderate and 3—intense.

#### Obtaining the popliteal lymph node cells

The popliteal draining lymph node was removed, weighed and processed in 1 mL sterile RPMI medium.

#### Leukocyte count

The cell suspensions obtained from the synovial lavage and lymph node were added to a crystal violet solution (0.05% and acetic acid to 30%) at a ratio of 9:1 (dye: cells). The cells were then quantified in a Neubauer chamber using a light optical microscope.

### Molecular docking

The interaction between ascaridole, the main monoterpene found in the *C*. *ambrosioides* species, and the pain receptor NMDA was analyzed using molecular docking. The ascaridole structure was obtained from the PubChem database—Code 10545. The terpene geometry was optimized in a vacuum at medium level of Density Functional Theory (DFT) to obtain the atomic and molecular electronic properties that are correlated with biological activity, using a B3LYP hybrid functional with base set 6–31++G (d, p) using *Gaussian software* 09 (*Frisch*, *Wallingford*, *CT*, *USA*). Vibrational frequencies were calculated from analytic second derivatives to check the minimum on the potential energy surface. The NMDA receptor structure used was obtained from *Rattus norvegicus* and was available in the Protein Data Bank (code 4NF5).

A protein with a crystallographed glutamatergic receptor was used in the molecular docking assays with NMDA receptors. This protein consists of two chains (A and B) that differ in their amino acid compositions. The ligands present therein were removed, and one docking was performed separately in each chain.

The AutoDock 4.2 package [22] was used for molecular docking calculations. The AutoDock Tools module was used to prepare and analyze computer simulations. *Gasteiger* loads and polar hydrogen necessary for the potential calculations were added considering the target structure, with the water molecules removed. The *Gasteiger* loads were also assigned to the ligands, and the non-polar hydrogens were suppressed. The rotatable interactions of each ligand were defined automatically. AutoDock requires pre-calculated three-dimensional maps, arranged in a box composed of a three-dimensional grid map in a defined region of the macromolecule (target site). AutoGrid 4.0 software was used to generate the maps for the ligands. The box was placed in the catalytic region of the receptor, centered on the portion of the amino acids Tyr143 (Chain A) and Arg121 (Chain B), identified as the active site of the NMDA receptor. The dimensions of the box in the X-, Y- and Z-axes were 60 Å x 60 Å x 60 Å, respectively, with a spacing of 0.375 Å [23].

The Lamarckian genetic algorithm (LGA) [24] was chosen to search for the best conformations, with 100 runs for each ligand (genetic algorithm with local search). During the search process, the NMDA receptor structure was kept rigid while the ligands were kept flexible. The initial population was maintained at 150, and the search was performed using random initial conformations. The complex with the best result was identified based on inhibition constants and residues that best interacted with the ligand. Molecular analyses and complex representations were obtained using the UCSF Chimera package, and representations of hydrogen and hydrophobic interactions were generated using LigPlot++ software [25–26].

### Statistical analysis

Comparison of the means of the different experimental groups was performed using the Student *t* test or analysis of variance (One-way ANOVA) followed by the Tukey test, after normality tests. Bivariate analysis of variance (Two-way ANOVA) was used when evaluating two sources of variability. A p-value of <0.05 was considered to indicate significance, and the data obtained were analyzed using GraphPadInStat® software (GraphPad Software, San Diego, CA, USA).

## Results

### Evaluation of joint inflammation

**Fig 1:**
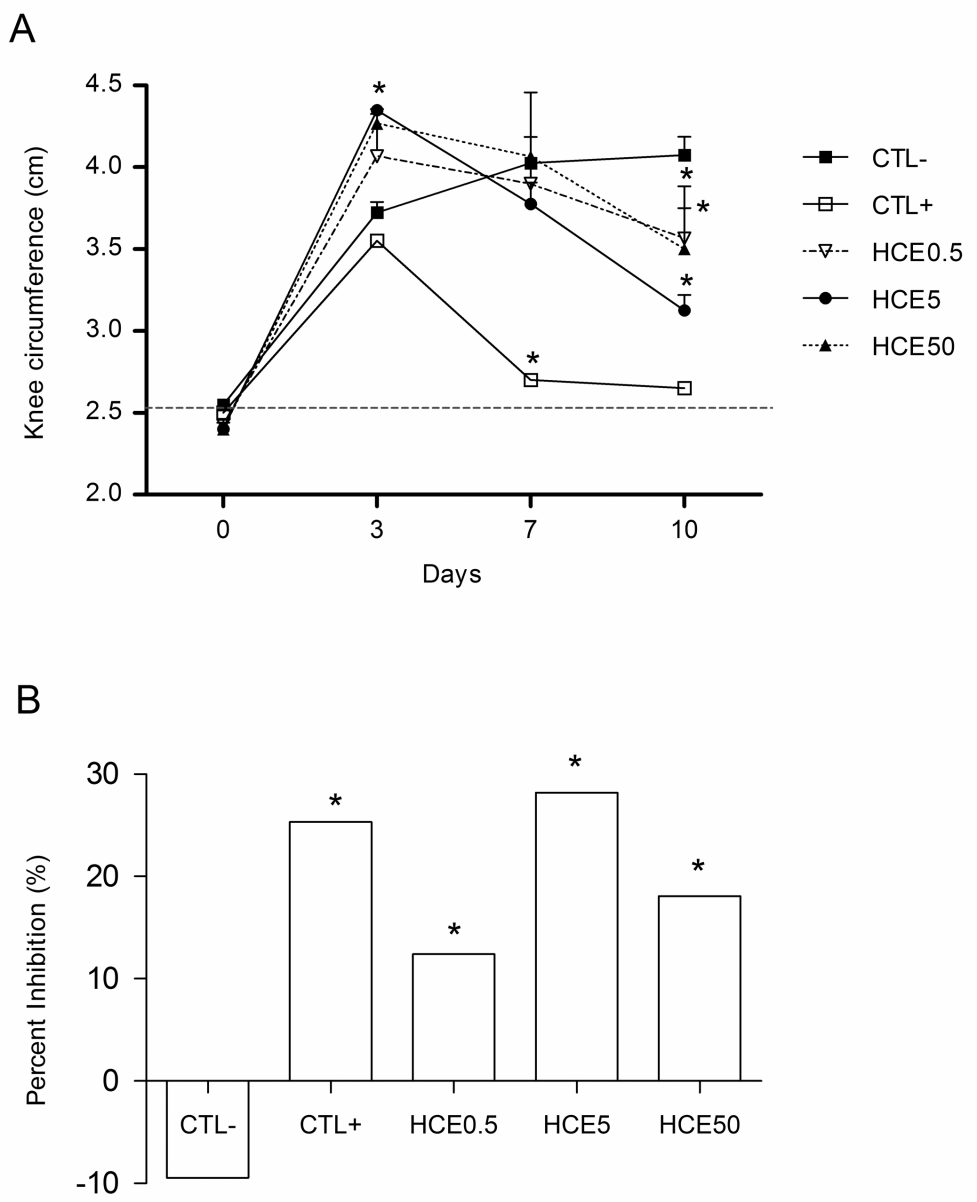
Measurement of knee with OA from animals treated with *Chenopodium ambrosioides* HCE. OA was induced by MIA injection (8 mg/kg) in Wistar rat knees. After six hours, the animals received, by oral route, saline (CTL-), *Maxicam plus* (CTL+) or *Chenopodium ambrosioides* HCE at doses of 0.5, 5 and 50 mg/kg (HCE0.5, HCE5 and HCE50, respectively). After three, seven and ten days, the knee diameters of the animals were measured for indirect evaluation of inflammation. A shows the kinetics of inflammation, and B shows the percentage inhibition of inflammation on day ten compared to day three. The data are represented as the means ± standard errors of the mean. The dashed red line represents the Clean group (without OA and untreated). *p<0.05 compared to CTL- group.

The animals showed increased knee diameter on the third day after the MIA injection, that was more evident in the groups treated with HCE. On the seventh day, there was an increase in knee diameter in the CTL- group and reductions in the other groups. The diameter reduction was statistically significant in the CTL+ group (p<0.05). On the tenth day, all treated groups showed smaller knee diameter when compared to CTL- (Fig 1A). When the percentage inhibition of the treatments was analyzedrelative to the third day, all groups had decreased the knee diameter, except CTL-. This inhibition was more evident in theHCE5 group, which was similar to CTL+ (Fig 1B).

### Histopathological evaluation

Histopathological analysis of the synovial membrane revealed that the inflammatory cell infiltrate was predominantly mononuclear. This infiltration was lower only in the HCE5 group compared to the CTL- group. No significant differences were observed between groups in terms of edema (Table 1).

**Table 1:** Histopathological analysis of the synovial membrane from animals with MIA- induced OA and treated with *Chenopodium ambrosioides* HCE.

	Clean	CTL-	CTL+	HCE0.5	HCE5	HCE50
Inflammatory infiltrate	1.0±0.0^a^	2.0±0.0	2.0±0.5	3.0 ± 1.1	1.0 ± 0.5 *	2.0 ± 0.5
Edema	0.0 ± 0.0	0.0 ± 1.5	0.5 ± 0.9	2.0 ± 1.1	0.0 ± 0.5	0.0 ± 0.5

^a^ Mean ±S.E.M.of following scores: 0—absent, 1—light, 2—moderate and 3 –intense.

* p ≤ 0.05 when compared to the CTL- group.

### Leukocyte counts of synovial lavage and popliteal lymph node

Synovial lavage cell counts revealed that among the groups treated with HCE, only the HCE5 group had a reduced number of inflammatory cells when compared to the CTL- group. With regard to popliteal lymph node cell count, only the HCE5 group showed an increase in cell numbers when compared to CTL- (Table 2).

**Table 2:** Count of synovial and popliteal lymph node cells from animals with MIA-induced OA and treated with *Chenopodium ambrosioides* HCE.

	Clean	CTL-	CTL+	HCE0.5	HCE5	HCE50
Synovial Fluid	0.0 ± 0.0^a^	132.0 ± 54.2	35.3± 22.3	119.3 ± 56.0	22.7± 2.0*	100.3 ± 41.7
Lymph node	49.7 ± 10.1	171.3 ± 38.9	97.3 ± 61.5	122.0 ± 54.2	274.8 ± 99.9 *	257.0 ± 38.1

^a^ Mean ±S.E.M.

* p ≤0.05 when compared to the CTL- group.

### Evaluation of mechanical allodynia

**Fig 2:**
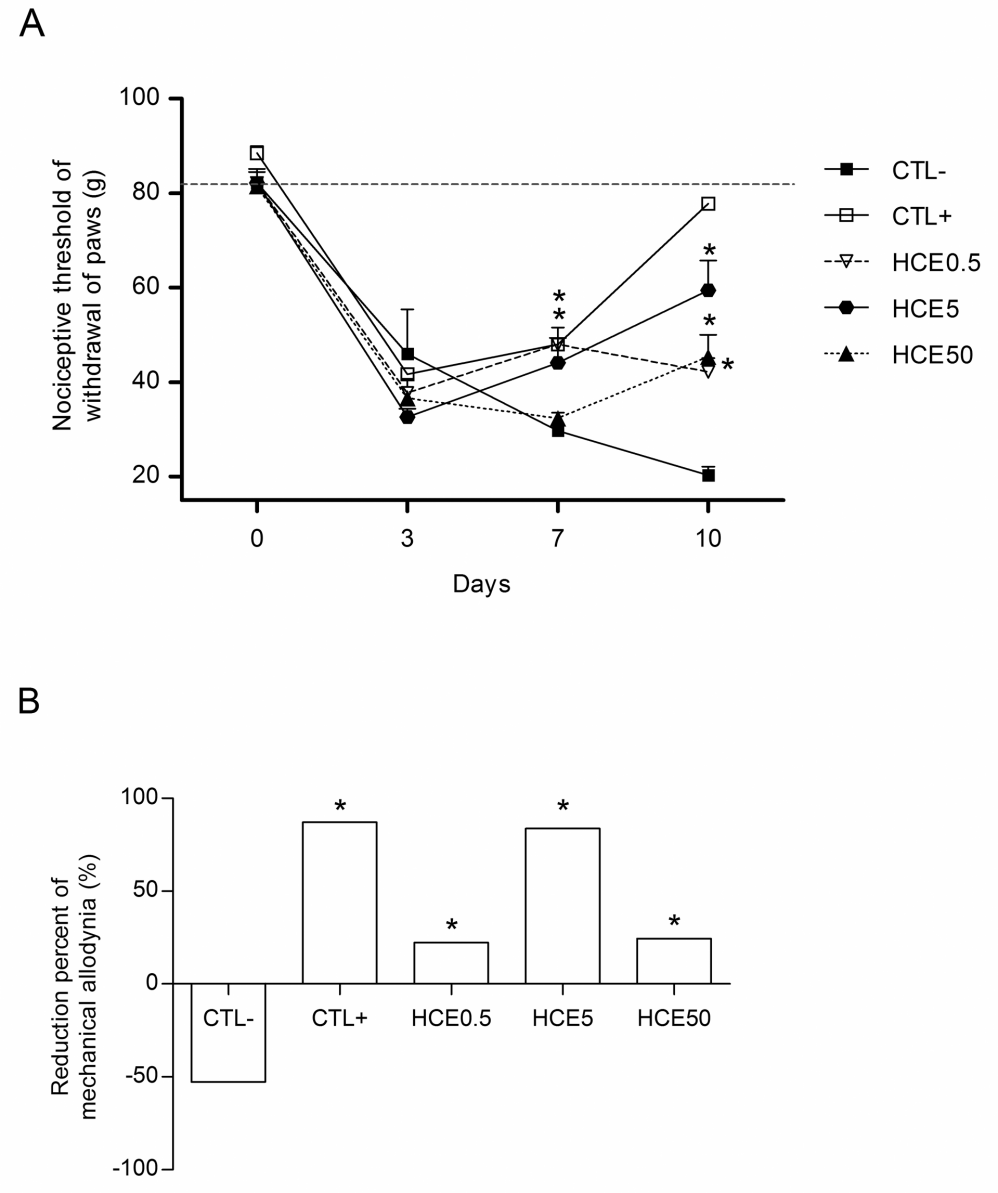
Evaluation of mechanical allodynia of animals with OA and treated with *Chenopodium ambrosioides* HCE. OA was induced by MIA injection (8 mg/kg) in Wistar rat knees. After six hours, the animals received, by oral route, saline (CTL-), *Maxicam plus* (CTL+) or *Chenopodium ambrosioides* HCE at doses of 0.5, 5 and 50 mg/kg (HCE0.5, HCE5 and HCE50, respectively). After three, seven and ten days, the nociceptive thresholds of animal leg withdrawal were measured for indirect evaluation using a *Von Frey* filament apparatus. A shows the kinetics of nociceptive threshold of leg withdrawal, and B shows the percentage reduction in mechanical allodynia on day ten relative to day three. The data are represented as the means± standard errors of the mean. The dashed red line represents the Clean group (without OA and untreated). *p<0.05 compared to CTL- group.

The animals exhibited decreases in the nociceptive leg withdrawal threshold on the third day after the MIA injection, characterizing mechanical allodynia. These decreases occurred in all experimental groups. On the seventh day, there were decreases in the thresholds of the CTL- and HCE50 groups and increases in the thresholds in the HCE0.5, HCE5 and CTL+ groups. On the tenth day, all groups treated with HCE showed improvement in the intensity of mechanical allodynia, as evidenced by increases in the nociceptive threshold, which was also significant in the CTL+ group when compared to CTL- (Fig 2A). When the percentage reductions in mechanical allodynia of HCE treatments were analyzed in relation to the third day, all groups, except CTL-, presented increases in their nociceptive thresholds, improving induced mechanical allodynia. This improvement was more evident in the HCE5 group, which was similar to CTL+ (Fig 2B).

### Evaluation of mechanical hyperalgesia

**Fig 3:**
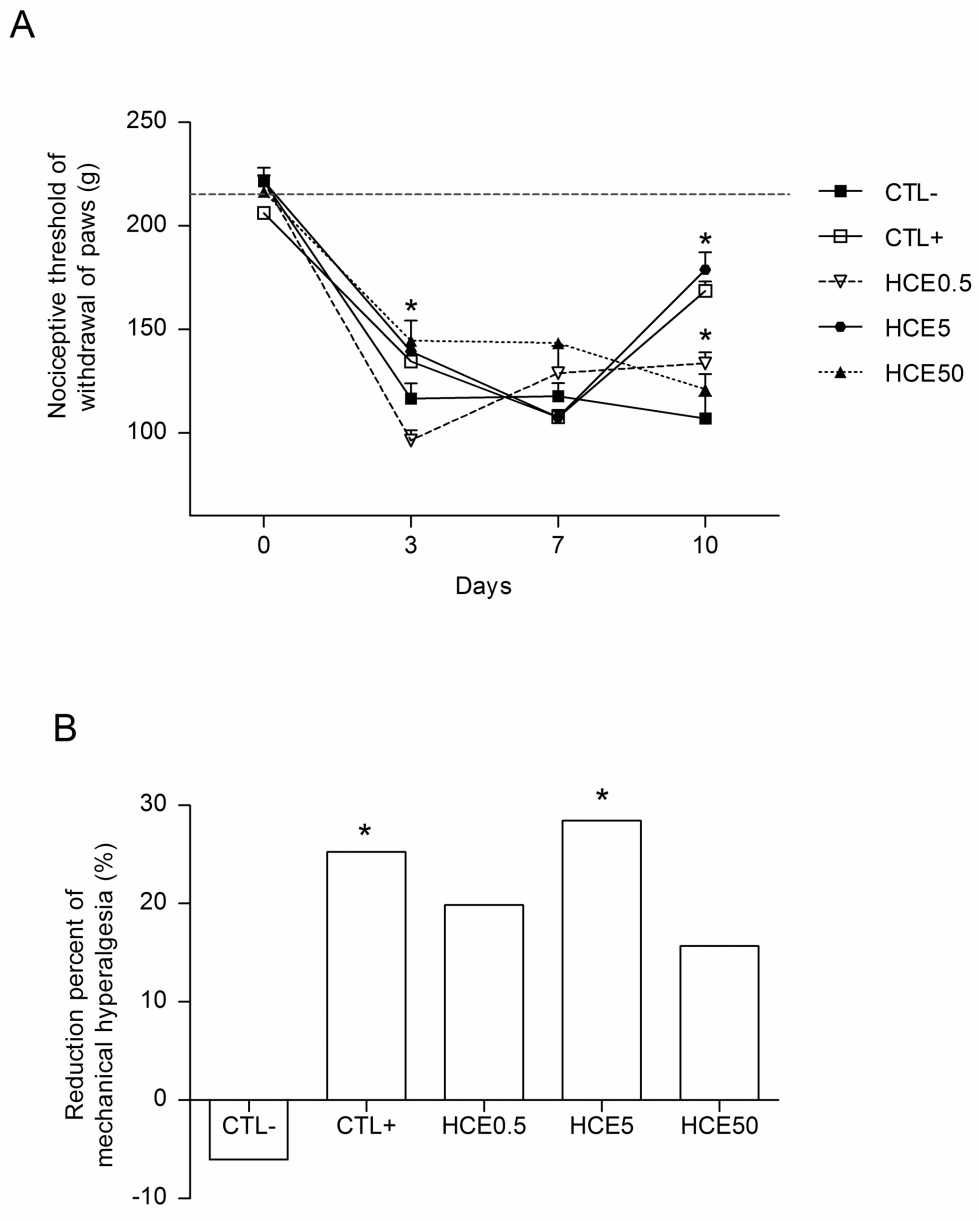
Evaluation of the mechanical hyperalgesia of animals with OA and treated with *Chenopodium ambrosioides* HCE. OA was induced by MIA injection (8 mg/kg) in Wistar rat knees. After six hours, the animals received, by oral route, saline (CTL-), *Maxicam plus* (CTL+) or *Chenopodium ambrosioides* HCE at doses of 0.5, 5 and 50 mg/kg (HCE0.5, HCE5 and HCE50, respectively). After three, seven and ten days, the nociceptive thresholds of animal leg withdrawal were measured for indirect evaluation using a *Randall-Selitto* apparatus. A shows the kinetics of nociceptive threshold of leg withdrawal, and B shows the percentage reduction in mechanical hyperalgesia on day ten relative to day three. The data are represented as the means± standard errors of the mean. The dashed red line represents the Clean group (without OA and untreated). *p<0.05 compared to CTL- group.

The animals also exhibited decreased leg withdrawal nociceptive thresholds on the third day after the MIA injection. These decreases occurred in all groups treated with HCE, in addition to the CTL+ and CTL-. On the tenth day of the experiment, all treated groups except for HCE50 showed increases in the nociceptive threshold, with significant improvements in mechanical hyperalgesia compared to the CTL- group (Fig 3A). When the percentage reductions of mechanical hyperalgesia of the treatments were analyzed relative to the third day, all groups except the CTL- showed improvements in induced mechanical hyperalgesia, which were significant for the HCE5 and CTL+ groups. The improvement in the inhibition percentage was more evident in the HCE5 group, which showed results similar to CTL+ (Fig 3B).

### Evaluation of motor activity

**Fig 4:**
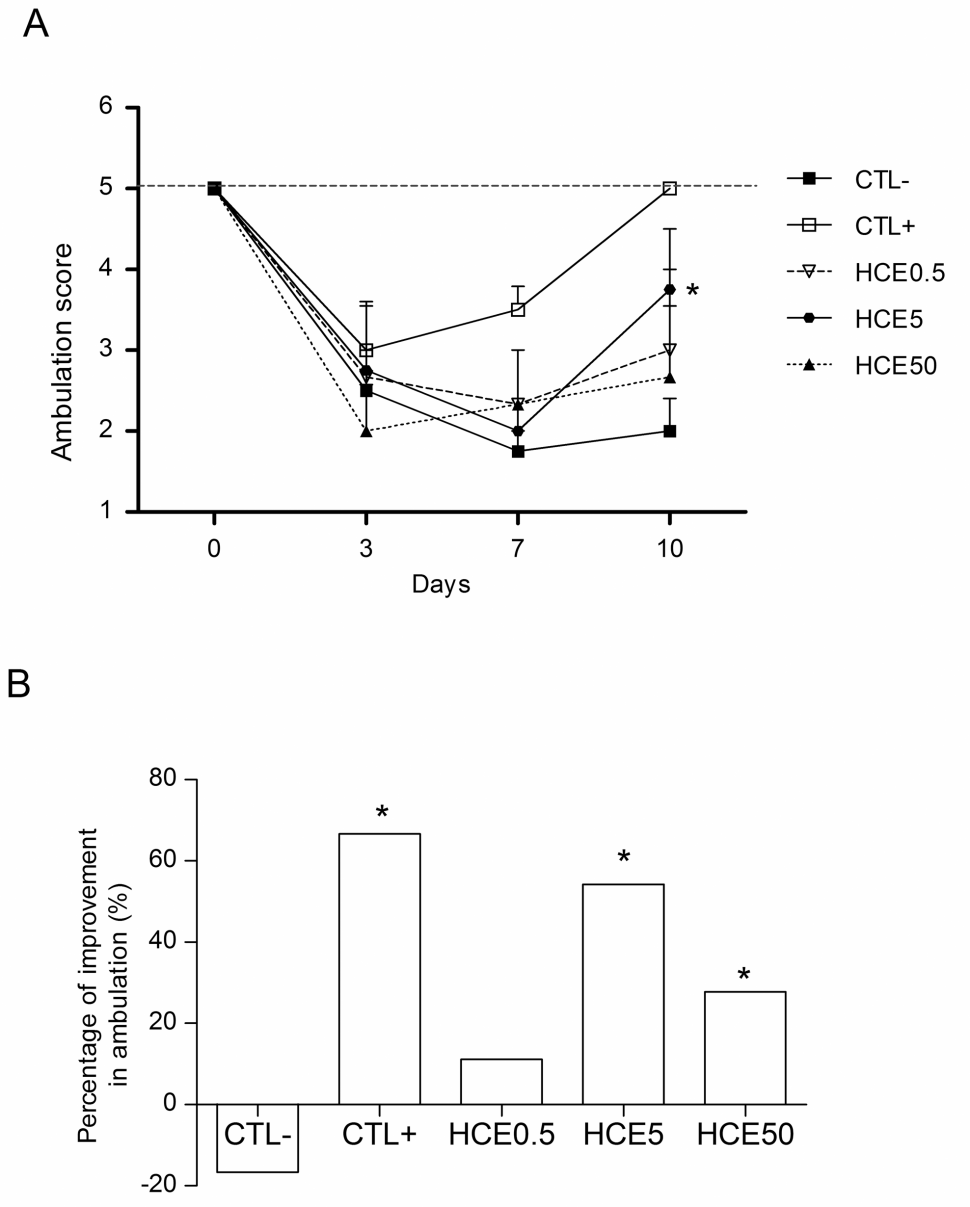
Evaluation of the motor activity of animals with OA and treated with *Chenopodium ambrosioides* HCE. OA was induced by MIA injection (8 mg/kg) in Wistar rat knees. After six hours, the animals received, by oral route, saline (CTL-), *Maxicam plus* (CTL+) or *Chenopodium ambrosioides* HCE at doses of 0.5, 5 and 50 mg/kg (HCE0.5, HCE5 and HCE50, respectively). After three, seven and ten days, the motor activity of the animals was measured to evaluate the walking score using a *Rotarod* device. A shows the kinetics of the animals' walking score, and B shows the percentage improvement in walking on day ten compared to day three. The data are represented as the means± standard errors of the mean. The dashed red line represents the Clean group (without OA and untreated). *p<0.05 compared to CTL- group.

Three days after the MIA injection, the animals showed decreases in the forced walking score, as expected. On the seventh day, the CTL-, HCE0.5 and HCE5 groups still showed decreases in the walking score, while the HCE50 and CTL+ groups showed improvements in walking, in the latter case a significant improvement. On the tenth day, all groups treated with HCE had increased scores, with improved walking; however, only the HCE5 group had a significant increase. A similar result was noted for the CTL+ group compared to the CTL- group (Fig 4A). When the percentage walking improvement of the treatments was studied relative to the third day, all treatment groups showed improvements in walking percentage, with the exception of HCE0.5. The improvement was most evident in the HCE5 group, which was close to the CTL+ value (Fig 4B).

### *In silico* analysis of ascaridole–NMDA receptor interaction

Due to the promising results presented, showing an analgesic effect of *C*. *ambrosioides* HCE and knowing that ascaridole is present in large quantities in its extract [12], and given that ascaridole has been cited as potentially involved in inflammation [10], we attempted to elucidate whether this substance was responsible for this effect by bonding it with the pain receptor (NMDA).

**Fig 5:**
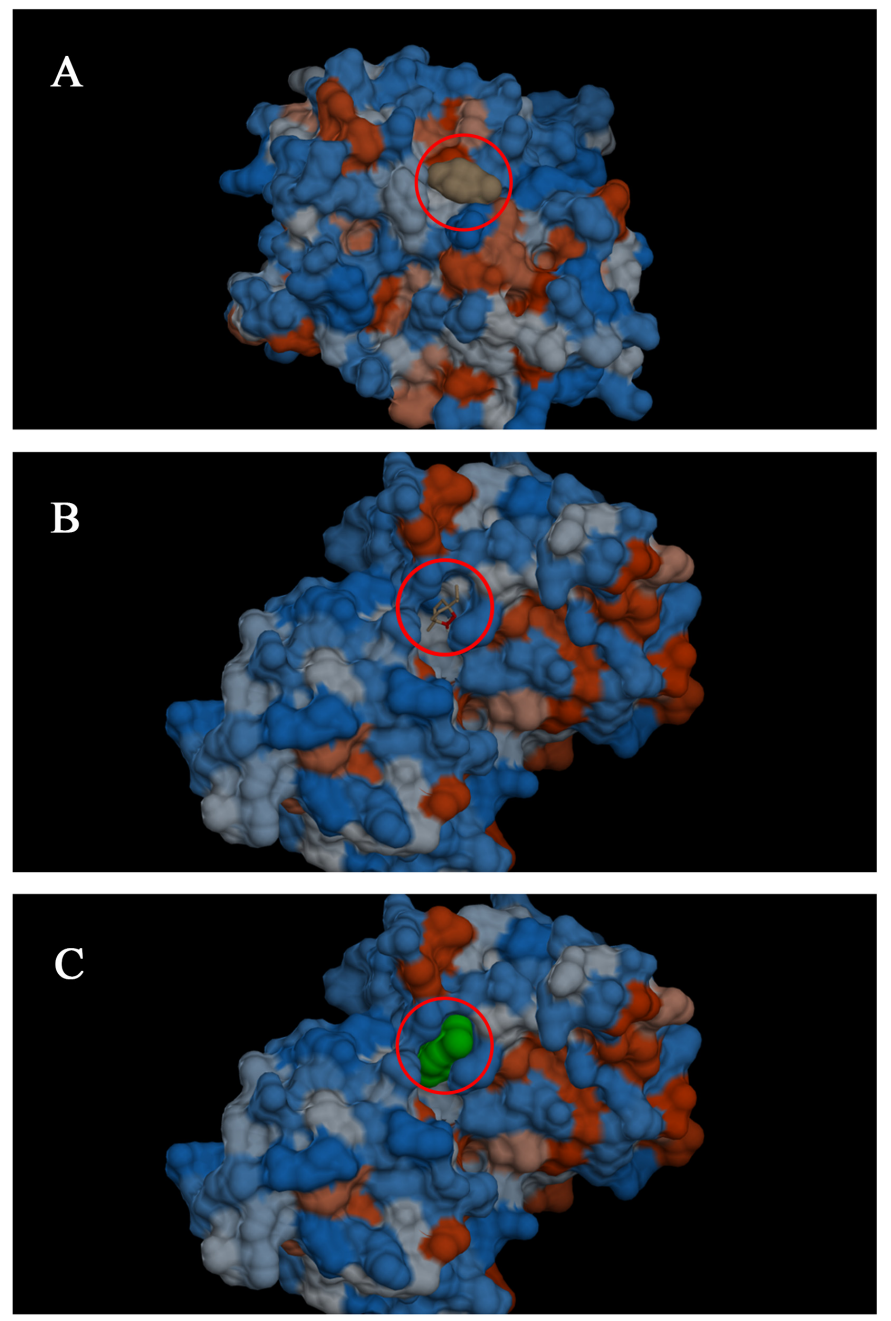
Three-dimensional image of the molecular docking of the interaction of ascaridole with chains A and B of the NMDA receptor. A shows chain A with ascaridole bound to the receptor; the terpene is represented in brown (red circle) in *surface* mode. B shows chain B with ascaridole bound to the receptor; the terpene is represented in its normal conformation (red circle). C shows the B chain with ascaridole bound to the receptor; the terpene is represented in green (red circle) in a different viewing mode (*surface*).

**Fig 6:**
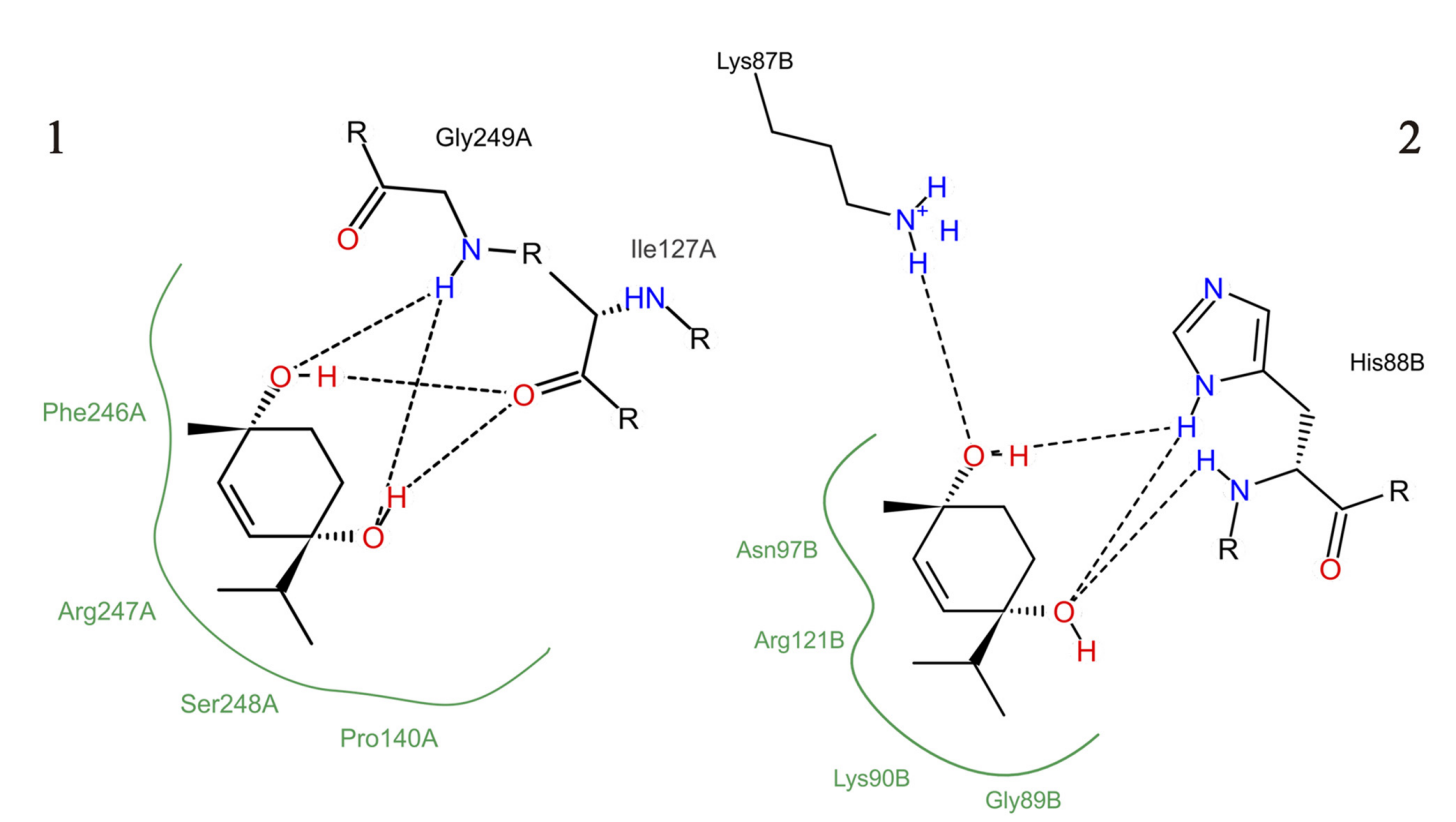
Molecular interaction of ascaridole with NMDA chains A (1) and B (2).

*In silico* analysis indicated that ascaridole can bond to both chain A (Fig 5A) and chain B (Fig 5B and 5C) of the glutamatergic receptor, both containing NMDA receptors. The ascaridole interaction at the active site of NMDA chains A and B featured negative binding energies (-5.19 kcal mol^-1^for chain A and -5.36 kcal mol^-1^ for chain B) (Fig 6). Hydrogen bonds (black dashed lines) and hydrophobic interactions were observed.

## Discussion

The use of plants as an alternative and/or complementary resource in disease treatment has grown in recent years, and the search for drugs that relieve pain symptoms has become increasingly intense [2]. However, few plants are used for OA treatment, and although there is no drug that can cure this disease, some, such as *C*. *ambrosioides*, could be used to alleviate the symptoms of pain and inflammation, since the results of this study clearly demonstrate its effects on osteoarthritis-related pain and inflammation.

The treatment with *C*. *ambrosioides* HCE reduced the knees diameter, what was more evident in HCE5 group. This parameter has been used as an inference of edema formation. However, when the synovial cartilage was microscopically observed, there was no edema inhibition. This result suggests that the knee diameter reduction was due to inhibition of inflammatory cells recruitment to synovia and not to edema. In fact, the HCE5 group also showed a decrease in synovial lavage cells that was consistent with inhibition of the inflammatory infiltrate in the synovia. It should be noted that the inflammatory infiltrate observed was predominantly mononuclear, indicating that lymphocytes may play an important role in this model.

Our group using infectious inflammation [6], carrageenan-induced edema, cotton pellet granuloma, and cyclophosphamide-induced cystitis models has already discussed this anti-inflammatory effect of oral treatment with HCE. In these previous results we also verified that the 5mg/Kg doses is the most efficient in inhibit different kinds of inflammatory reaction. This fact can be related to the pharmacokinetics and pharmacodynamics of the HCE, but also to its toxicity. Our group showed that the sub chronic treatments with HCE at 5mg/Kg are totally safe. However, the dose of 50 mg/Kg showed punctual alterations in mice when used sub chronically [27], what was more evident in chronic use.

To investigate whether the inhibitory effect of inflammatory infiltrate was associated with a reduction in the secretion of pro-inflammatory cytokines, a measurement of cytokines from the synovial lavage of the animal’s knee was taken. However, no significant cytokine concentrations were detected in the lavage (data not shown).However, the HCE5 group had an increased number of draining lymph node cells, suggesting that the reduction in inflammatory infiltrate in synovial fluid was due to the maintenance of lymphocytes in the lymph node. It was not possible to investigate the kind of lymphocyte that was proliferating in the lymph node, but it is reasonable to suppose that regulatory lymphocytes or also M2 macrophage could be activated by HCE treatment and participating in both the regulation of inflammation and tissue repair [28,29].

Considering the significant inflammatory reduction observed in the knees, it was investigated whether the inhibition of inflammation could also reflect in the decreasing of pain associated to OA. At the end of treatment, the improvement of allodynia was more evident in the HCE5 group, in which a decrease of approximately 80% in nociceptive threshold was observed on the last day of the experiment. This reduction was also very close to that observed in the positive control group (86%). This result is in accordance with other authors [30,31] that observed this allodynia reduction after treatment with injectable lacosamide, a drug used for the treatment of epilepsy, or type II collagen, respectively.

The treatment with *C*. *ambrosioides* HCE was also able to reduce the intensity of mechanical hyperalgesia through the end of experiment. The results showed a reduction of 30% in mechanical hyperalgesia in the HCE5 group, which was similar to the positive control group, which showed a reduction of 26%. Our group has previously shown the antinociceptive effect of oral treatment with *C*. *ambrosioides* HCE using acetic acid-induced abdominal contortions test [8].

Altogether, the results demonstrated that *C*. *ambrosioides* HCE had anti-inflammatory and antinociceptive effects, which means that it is similar to drugs already used therapeutically. One example is diclofenac, a non-steroidal anti-inflammatory that reduced the intensity of mechanical hyperalgesia [32].

Based on the fact that HCE has an antinociceptive action, we investigated whether the motor ability would be also recovered. In fact, the walking scores of animals treated with HCE increased until the end of treatment, however, only the HCE5 group showed a significant increase in the walking ability, on the tenth day of the experiment. The fact that HCE improved walking in animals with MIA-induced OA demonstrates that the extract acts directly on knee inflammation, confirming the results of the work mentioned above and suggesting an antinociceptive response, which leads to an improvement in walking. Yassin and collaborators [33] obtained similar results using topical treatment with an indomethacin gel, suggesting that the anti-inflammatory effect is intrinsically related to the decreasing of pain and, consequently, the recovering of motor ability.

The significant differences observed between the HCE-treated and control groups in clinical evaluation tests could be explained by a possible antagonistic effect of the substances present in the *C*. *ambrosioides* extract on pain receptors, such as NMDA receptor, which is related to chronic pain. It was shown here that ascaridole binds to the NMDA receptors. This result suggests that HCE could act as antagonist of NMDA receptor what can explains the decreasing of inflammation and pain by *C*. *ambrosioides*. The HCE could acts in a similar way as ketamine, which has been identified as a potent NMDA antagonist [34].Other NMDA antagonist is Magnesium sulfate (MgSO_4_), which changes the course of OA induced in animals by reducing nociception, chondrocyte apoptosis and the expression of the NR1 receptor, an NMDA subtype that is related to the prevention of cartilage damage [35].

To date, no studies have been found in the scientific literature that have analyzed the relationship of ascaridole or any other substance from *C*. *ambrosioides* HCE with the NMDA receptor. Furthermore, we suggest that antagonism of this receptor is one of the probable mechanisms by which HCE attenuates pain in animals, with possible reduction of central sensitization and receptor expression.

Other studies analyzing the expression of these receptors in OA are necessary to confirm the findings of this work; however, it seems clear that there is a relationship between ascaridole and NMDA, and this relationship may be the key to a new way of treating OA, using plant species showing promising results for the production of new drugs.

## Conclusion

Oral treatment with *C*. *ambrosioides* HCE inhibited synovial inflammation, modulated lymphocyte proliferation and decreased the pain *In silico* analysis showed that ascaridole is an NMDA receptor antagonist, what could explain its effect on pain reduction.

## Supporting Information

S1 Fig Measurement of knee with OA from animals treated with *Chenopodium ambrosioides* HCE. OA was induced by MIA injection (8 mg/kg) in Wistar rat knees. After six hours, the animals received, by oral route, saline (CTL-), *Maxicam plus* (CTL+) or *Chenopodium ambrosioides* HCE at doses of 0.5, 5 and 50 mg/kg (HCE0.5, HCE5 and HCE50, respectively). After three, seven and ten days, the knee diameters of the animals were measured for indirect evaluation of inflammation. A shows the kinetics of inflammation, and B shows the percentage inhibition of inflammation on day ten compared to day three. The data are represented as the means ± standard errors of the mean. The dashed red line represents the Clean group (without OA and untreated). *p<0.05 compared to CTL- group.
(XLSX)

S2 Fig Evaluation of mechanical allodynia of animals with OA and treated with *Chenopodium ambrosioides* HCE. OA was induced by MIA injection (8 mg/kg) in Wistar rat knees. After six hours, the animals received, by oral route, saline (CTL-), *Maxicam plus* (CTL+) or *Chenopodium ambrosioides* HCE at doses of 0.5, 5 and 50 mg/kg (HCE0.5, HCE5 and HCE50, respectively). After three, seven and ten days, the nociceptive thresholds of animal leg withdrawal were measured for indirect evaluation using a *Von Frey* filament apparatus. A shows the kinetics of nociceptive threshold of leg withdrawal, and B shows the percentage reduction in mechanical allodynia on day ten relative to day three. The data are represented as the means± standard errors of the mean. The dashed red line represents the Clean group (without OA and untreated). *p<0.05 compared to CTL- group.
(XLSX)

S3 Fig Evaluation of the mechanical hyperalgesia of animals with OA and treated with *Chenopodium ambrosioides* HCE. OA was induced by MIA injection (8 mg/kg) in Wistar rat knees. After six hours, the animals received, by oral route, saline (CTL-), *Maxicam plus* (CTL+) or *Chenopodium ambrosioides* HCE at doses of 0.5, 5 and 50 mg/kg (HCE0.5, HCE5 and HCE50, respectively). After three, seven and ten days, the nociceptive thresholds of animal leg withdrawal were measured for indirect evaluation using a *Randall-Selitto* apparatus. A shows the kinetics of nociceptive threshold of leg withdrawal, and B shows the percentage reduction in mechanical hyperalgesia on day ten relative to day three. The data are represented as the means± standard errors of the mean. The dashed red line represents the Clean group (without OA and untreated). *p<0.05 compared to CTL- group.
(XLSX)

S4 Fig Evaluation of the motor activity of animals with OA and treated with *Chenopodium ambrosioides* HCE. OA was induced by MIA injection (8 mg/kg) in Wistar rat knees. After six hours, the animals received, by oral route, saline (CTL-), *Maxicam plus* (CTL+) or *Chenopodium ambrosioides* HCE at doses of 0.5, 5 and 50 mg/kg (HCE0.5, HCE5 and HCE50, respectively). After three, seven and ten days, the motor activity of the animals was measured to evaluate the walking score using a *Rotarod* device. A shows the kinetics of the animals' walking score, and B shows the percentage improvement in walking on day ten compared to day three. The data are represented as the means± standard errors of the mean. The dashed red line represents the Clean group (without OA and untreated). *p<0.05 compared to CTL- group.
(XLSX)

S1 Table Histopathological analysis of the synovial membrane from animals with MIA- induced OA and treated with *Chenopodium ambrosioides* HCE. (XLSX)

S2 Table Count of synovial and popliteal lymph node cells from animals with MIA-induced OA and treated with *Chenopodium ambrosioides* HCE. (XLSX)

## References

[1] Kumar R, Mishra AK, Dubey NK, Tripathi YB. Evaluation of *Chenopodium ambrosioides* oil as a potential source of antifungal, antiaflatoxigenic and antioxidant activity. Int J Food Microbiol. 2007; 115: 159–164. 17174000 10.1016/j.ijfoodmicro.2006.10.017

[2] Lorenzi H, Matos FJA. Plantas Medicinais no Brasil: nativas e exóticas. Nova Odessa, Plantarum 2002.

[3] Dembitsky V, Shkrob I, Hanus LO. Ascaridole and related peroxides from the genus *Chenopodium* . Biomedical Papers of the Medical Faculty of the University Palacký, Olomouc, Czechoslovakia, 2008; 152: 209–215. 10.5507/bp.2008.032 19219209

[4] Pinheiro Neto VF, Araujo BMA, Guerra PC, Borges MOR, Borges ACR. Efeitos do cataplasma das folhas de mastruz (*Chenopodium ambrosioides* L.) na reparação de tecidos moles e ósseo em rádio de coelhos. J Bras Fitomed, 2005; 3(2): 62–66.

[5] Nascimento FRF, Cruz GVB, Pereira PVS, Maciel MCG, Silva LA, Azevedo APS, et al Ascitic and solid Ehrlich tumor inhibition by Chenopodium ambrosioides L. treatment. J Life Sci. 2006; 78: 2650–2653. 10.1016/j.lfs.2005.10.006 16307762

[6] Patricio FJ, Costa GC, Pereira PVS, Aragao-Filho W, Sousa SM, Frazao JB, et al Efficacy of the intralesional treatment with *Chenopodium ambrosioides* in the murine infection by *Leishmania amazonensis* . J Ethnopharmacol. 2008; 115: 313–319. 18035510 10.1016/j.jep.2007.10.009

[7] Monzote L, Gille L, Scull R, Setzer WN, Steinbauer S, Garcia M, et al Combinations of ascaridole, carvacrol and caryophyllene oxide against *Leishmania* . Acta trop. 2015–17; 145: 31–38. 25697866 10.1016/j.actatropica.2015.02.002

[8] Sousa LHA, Rios CEP, Assunção AKM, Fialho EMS, Costa GC, Nascimento FRF. Avaliação da ação analgésica do extrato hidroalcoólico de *Chenopodium ambrosioides* L. em ensaios pré-clínicos. Rev Ciênc Saude. 2012; 14 (1).

[9] Trivellato-Grassi L, Malheiros A, Meyre-Silva C, Buss ZS, Monguilhot ED, Frode TS, et al From popular use to pharmacological validation: A study of the anti-inflammatory, anti-nociceptive and healing effects of *Chenopodium ambrosioides* extract. J Ethnopharmacol. 2013; 145: 127–138. 10.1016/j.jep.2012.10.040 23123797

[10] Ye H, Liu Y, Li N, Yu J, Cheng H, Li J, et al Anti-*Helicobacter pylori* activities of *Chenopodium ambrosioides* L. *in vitro* and *in vivo* . World J Gastroenterol. 2015; 21 (14): 4178–4183. 10.3748/wjg.v21.i14.4178 25892867 PMC4394078

[11] Olajide OA, Awe SO, Makinde JK. Pharmacological screening of the methanolic extract of *Chenopodium ambrosioides* . Fitoterapia. 1997; 68: 529–532.

[12] Ibironke GF, Ajiboye KI. Studies of anti-inflammatory and analgesic properties of *Chenopodium ambrosioides* leaf extract in rats. Int J Pharmacol. 2007; 3 (1): 111–115.

[13] Kitchen DB, Decornez H, Furr J, Bajorath J. Docking and soring in virtual screening for druga discovery: methods and applications. Drug discovery. 2004; 3: 935–949. 15520816 10.1038/nrd1549

[14] Ishizuka P, Garcia JBS, Sakata RK, Issy AM, Mulich SL. Assessment of Oral S(+) Ketamine Associated with Morphine for the Treatment of Oncologic. Rev Bras Anestesiol. 2007; 57 (1): 19–31. 19468615 10.1590/s0034-70942007000100003

[15] Neiva VA, Amaral FMM, Cartagenes MSS, Moraes-Coutinho DF, Nascimento FRF, Reis VA, et al Estudos pré-clínicos de atividade giardicida de *Chenopodium ambrosioides* L. e a padronização dos extratos na pesquisa e desenvolvimento de fitoterápicos. Rev Ciênc Saúde. 2011; 2: 155–165.

[16] Matos FJA. Introdução a fitoquímica experimental 2° Ed. Fortaleza Edições UFC: 1997.

[17] Fernihough J, Gentry C, Malcangio M, Fox A, Rediske J, Pellas T, et al Pain related behaviour in two models of osteoarthritis in the rat knee. Pain. 2004; 112: 83–93. 15494188 10.1016/j.pain.2004.08.004

[18] Moller KA, Johansson B, Berge OG. Assessing mechanical allodynia in the rat paw with a new electronic algometer. J Neurosci Methods. 1998; 84: 41–47. 9821632 10.1016/s0165-0270(98)00083-1

[19] Randall LO, Selitto JJ. A method for measurement of analgesic activity on inflamed tissue. Arch Int Pharmacodyn Ther. 1957; 111: 409–419. 13471093

[20] Castro RR, Cunha FQ, Silva FS Junior, Rocha FACA. Quantitative approach to measure joint pain in experimental osteoarthritis-evidence for a role for nitric oxide. Osteoarthriitis Cartiliage. 2008; 14: 769–768. 10.1016/j.joca.2006.01.013 16580848

[21] Rocha FAC, Rocha JCS, Peixoto MEB, Jancar S, Cunha FQ, Ribeiro RA. Effect of nitric oxide synthase in articular inflammatory pain and cellular influx of zymosan-induced arthritis in rats. Rev Bras Reumatol. 2003; 43: 206–217.

[22] Goodsell DS, Morris GM, Olson AJ. Automated docking of flexible ligands: applications of AutoDock. J Mol Recognit. 1996; 9: 1–5. 8723313 10.1002/(sici)1099-1352(199601)9:1<1::aid-jmr241>3.0.co;2-6

[23] Ramos RM, Perez JM, Baptista LA, Amorim HLN. Interaction of wild type, G68R and L125M isoforms of the arylamine-N-acetyltransferase from *Mycobacterium tuberculosis* with isoniazid: a computational study on a new possible mechanism of resistance. J Mol Model. 2012; 18: 4013–4024. 10.1007/s00894-012-1383-6 22460521

[24] Morris GM, Goodsell DS, Halliday RS, Huey R, Hart WE, Belew RK, et al Automated docking using a lamarckian genetic algorithm and empirical binding free energy function. J Comput Chem. 19; 1639–1662.

[25] Laskowski RA, Swindells MB. LigPlot+: multiple ligand-protein interaction diagrams for drug discovery. J Chem Inf Model 2011; 51: 2778–2786. 10.1021/ci200227u 21919503

[26] Stierand K, Rarey M. Drawing the PDB: protein-ligand complexes in two dimensions. ACS Med Chem Lett. 2010; 1 (9): 540–545. 10.1021/ml100164p 24900245 PMC4007829

[27] Pereira WS, Ribeiro BP, Sousa AIP, Serra ICPB, Mattar NS, Fortes TS, et al Evaluation of the subchronic toxicity of oral treatment with Chenopodium ambrosioides in mice. J. Ethnopharmacol. 2010; 127: 602–605. 10.1016/j.jep.2009.12.018 20026398

[28] Liu G, Ma H, Qiu L, Li L, Cao Y, Ma J, et al Phenotypic and functional switch of macrophages induced by regulatory CD4+CD25+ T cells in mice. Immunol Cell Bio. 2011; 89: 130–142. 20514074 10.1038/icb.2010.70

[29] Martinez FO, Helming L, Milde R, Varin A, Melgert BN, Draijer C, et al Genetic programs expressed in resting and IL-4 alternatively activated mouse and human macrophages: similarities and differences. Blood. 2013; 121: 57–69. 10.1182/blood-2012-06-436212 23293084

[30] Rahman W, Dickenson A. Antinociceptive effects of lacosamide on spinal neuronal and behavioural measures of pain in a rat model of osteoarthritis. Arthritis Res Ther. 2014; 16: 509 10.1186/s13075-014-0509-x 25533381 PMC4308925

[31] Manelli LDC, Micheli L, Zanardelli M, Ghelardini C. Low dose native type II collagen prevents pain in a rat osteoarthritis model. BMC Musculoskelet Disord. 2013; 14: 228 10.1186/1471-2474-14-228 23915264 PMC3751133

[32] Goh JZ, Tang SN, Chiong HS, Yong YK, Zuraini A, Hakim MN. Evaluation of antinociceptive activity of nanoliposome-encapsulated and free-form diclofenac in rats and mice. Int J Nanomedicine. 2015; 10: 297–303. 10.2147/IJN.S75545 25678786 PMC4317161

[33] Yassin NZ, El-Shenawy S, Abdel-Rahman R, Yakoot M, Hassan M, Helmy S. Effect of a topical copper indomethacin gel on inflammatory parameters in a rat model of osteoarthritis. Drug Des Devel Ther. 2015; 9: 1491–1498. 10.2147/DDDT.S79957 25792809 PMC4362896

[34] Kiselycznyk C, Jury NJ, Halladay LR, Nakazawa K, Mishina M, Sprengel R, et al NMDA receptor subunits and associated signaling molecules mediating antidepressant-related effects of NMDA-GluN2B antagonism. Behav Brain Res. 2015; 287: 89–95. 10.1016/j.bbr.2015.03.023 25800971 PMC4425283

[35] Lee CH, Wen ZH, Chang YC, Huang SY, Tang CC, Chen WF, et al Intra-articular magnesium sulfate (MgSO4) reduces experimental osteoarthritis and nociception: association with attenuation of N-methyl-D-aspartate (NMDA) receptor subunit 1 phosphorylation and apoptosis in rat chondrocytes. Osteoarthritis Cartilage. 2009; 17: 1485–1493. 10.1016/j.joca.2009.05.006 19490963

